# DNA Sensors for the Detection of Mercury Ions

**DOI:** 10.3390/bios15050275

**Published:** 2025-04-29

**Authors:** Feng Li, Jinxing Lin, Eric Lichtfouse, Haifeng Qi, Lang Peng, Yangyang Yu, Li Gao

**Affiliations:** 1 Department of Thoracic Surgery, Affiliated Hospital of Jiangsu University, Zhenjiang 212013, China; lifengjs@163.com (F.L.); linjx1218@163.com (J.L.); 18796012875@163.com (H.Q.); 15330460812@163.com (L.P.); 2 International Research Center for Renewable Energy, State Key Laboratory of Multiphase Flow in Power Engineering, Xi’an Jiaotong University, No. 28 Xianning West Rd, Xi’an 710049, China; eric.lichtfouse@gmail.com; 3 Information Materials and Intelligent Sensing Laboratory of Anhui Province, Anhui University, Hefei 230039, China; 4 School of Life Sciences, Jiangsu University, Zhenjiang 212013, China; 5 School of Life Sciences, Qinghai Normal University, Xining 810008, China

**Keywords:** detection of Hg^2+^, DNA-based sensors, methods

## Abstract

Ecosystem pollution by mercury ions (Hg^2+^) is a major health concern, yet classical analytical methods for mercury analysis are limited. This paper reviews the advances in Hg^2+^ detection using DNA as recognition elements in the sensors. DNA as a recognition molecule is inexpensive, simple, and appropriate for real-time detection of Hg^2+^. This paper discusses the DNA-based sensors that were used for the detection of Hg^2+^. These can be carried out by electrochemistry, field effect transistors (FET), Raman spectroscopy, colorimetry, and fluorescence resonance energy transfer (FRET). The detection principles and the advantages of DNA in these sensors are also revealed. Finally, the paper provides an overview of prospects and potential challenges in the field.

## 1. Introduction

Humans and ecosystems face environmental pollution caused by heavy metals, as evidenced by incidents such as the Rhine pollution [1] and the Japanese Minamata disease [2], which resulted in severe health issues such as bone pain. The Rhine pollution incident comprised a 70-km-long pollution belt, killing all fish flowing through the river. The standard safe concentration of mercury in human blood is 1 µg/10 mL (5 × 10^−7^ M), and poisoning symptoms appear at 5~10 µg/10 mL (2.5 × 10^−6^ to 5 × 10^−6^ M). Lead, cadmium, mercury, and arsenic are refractory and accumulate in the biological chain, finally reaching toxic concentrations in the human body, causing damage to human health [3,4]. Mercury, arsenic, cadmium, copper, lead, tin, and manganese can cause irreversible damage to the human body if they exceed the standard value [5,6].

Hg^2+^ is a major threat to food safety, with potential contamination of food via multiple pathways, thus endangering human health through people’s dietary intake [7,8,9,10]. Hg^2+^ is easy to accumulate in the central nervous system of humans [11,12,13]. Ingestion of Hg^2+^ can cause lung injury, vomiting, diarrhea, nausea, movement disorders, as well as language and hearing impairment. Moreover, Hg^2+^ damages nerves and other organs and causes severe dysfunctions such as kidney and muscle issues, deformed limbs, paralysis, degeneration and necrosis of brain cells, difficulty in swallowing, and death [14,15,16,17,18]. The half-life of Hg^2+^ in the human body is rather long, approximately 70–80 days. Usually, Hg^2+^ is detected in blood or hair, which primarily reflects only organic mercury exposure [19]. Aggregation of Hg^2+^ concentration usually occurs in the liver, brain, kidney, and placenta, especially in the peripheral nerves, fetal brain, and bone marrow [20]. The presence of Hg^2+^ in even trace amounts can cause serious and irreversible harm to the human body, leading to a range of symptoms, including low-grade fever, fatigue, dizziness, headaches, sleep disturbances, adrenaline fluctuations, neurodevelopmental issues, and neurodegenerative conditions [21]. Given these risks, it is crucial to develop reliable methods for detecting Hg^2+^ to safeguard both human health and the environment [22,23,24,25,26].

Traditional detection methods, such as mass spectrometry [27,28,29,30,31], have made significant progress in the detection of Hg^2+^. However, these often require complex equipment, are costly, and can be time-consuming [32]. A significant breakthrough appeared in 2004 when Ono et al. [33] revealed that Hg^2+^ could selectively bind to DNA sequences containing thymine bases, forming a stable T-Hg^2+^-T structure [34]. This unique interaction opened the door to a new class of detection methods that leverage the stability and selectivity of DNA for quick and cost-effective detection [35,36]. Hg^2+^ replaces the hydrogen bonds typically found in Watson-Crick T-A pairing, thereby stabilizing the DNA duplex or structural motifs. Density functional theory (DFT) calculations have elucidated the electronic properties and bonding nature of the T-Hg^2+^-T complex [37,38]. The specific interaction of mercury ions (Hg^2+^) with DNA is determined by several key physicochemical factors, distinguishing it from other metal ions. One crucial factor is the soft acid-soft base principle (HSAB theory), which explains why Hg^2+^, a soft Lewis acid, preferentially interacts with thymine’s nitrogen (N_3_) and oxygen (O_4_) atoms, which are soft Lewis bases. This results in the formation of a highly stable T-Hg^2+^-T base pair, where Hg^2+^ bridges two thymine residues, replacing conventional hydrogen bonds. The unique coordination chemistry of Hg^2+^ enables selective and strong binding, stabilizing DNA structures and making it an excellent target for DNA-based mercury sensors. Compared to other metal ions, Hg^2+^ exhibits distinct electronic and coordination properties. While silver ions (Ag^+^) also form metal-mediated base pairs (e.g., C-Ag^+^-C), their interaction mechanism and stability differ from Hg^2+^ [39]. Monovalent cations such as lithium (Li^+^) [40], sodium (Na^+^) [41], and potassium (K^+^) [42] primarily interact with the phosphate backbone of DNA, influencing its structure and stability rather than forming base pair-mediated complexes. Bivalent metals like copper (Cu^2+^) [43], magnesium (Mg^2+^) [44], and lead (Pb^2+^) [45] interact differently; for example, Cu^2+^ can intercalate into DNA, Mg^2+^ stabilizes DNA duplexes via electrostatic interactions, and Pb^2+^ exhibits toxic binding effects that distort DNA structure. Ding et al. used isothermal titration calorimetry experiments to find that DNA tended to make the link ring of the hairpin a segment of four or five bases in the process of hairpin folding from a random coil induced by the external environment [46,47]. However, most researchers have not optimized DNA sequences for Hg^2+^ detection.

DNA is usually stable, low-cost, easy to fix and regenerate, which has been widely used in the sensor field. Various sensors have been developed based on the DNA recognition principle, including electrochemical sensors, FET, Raman, colorimetry, and FRET. This article reviews DNA-based approaches for Hg^2+^ detection, focusing on mechanisms, applications, and real-time monitoring. Compared to published reviews on related topics, such as fluorescence-based techniques, nanozymes, and surface-enhanced Raman spectroscopy, our manuscript focuses on DNA-based sensors (Table 1). DNA-based sensors show significant advantages, including high specificity, low cost, and high sensitivity. Therefore, DNA-based sensors have a wide range of applications in Hg^2+^ detection.

## 2. Electrochemistry

**Figure 1:**
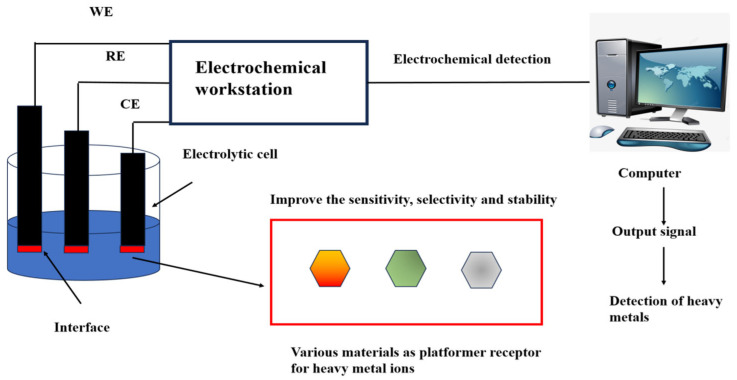
Heavy metal ions detection using electrochemical sensing. The working electrode, reference electrode, and counter electrode are inserted into an electrolytic cell containing a heavy metal. The heavy metal ion platform or receptor at the interface of the working electrode reacts with the heavy metal to produce a changing current signal. The electrochemistry workstation, consisting of three electrodes, receives the signal and transmits it to the computer for analysis and processing. The output signal materializes the data to detect heavy metals directly or indirectly. Working electrode (WE): The interface contains a variety of materials that act as platforms or receptors for heavy metal ions. This improves the sensitivity, selectivity, and stability in heavy metal detection. The reference electrode (RE) is an electrode used as a reference when measuring the potential of various electrodes. The counter electrode (CE) balances the current to ensure that the electrochemical reaction can continue. Reproduced from Ju et al. (2015) with permission of Elsevier [67].

Electrochemical analysis is a pivotal branch of sensor technology, renowned for its accurate detection results and highly adaptable in in-situ measurements [62]. The US Environmental Protection Agency recommends using electrochemical technology to detect Hg^2+^ [63]. Electrochemical sensors were first used in oxygen detection in the 1950s and have been continuously improved by researchers to detect various analytes. The combination of DNA and electrochemistry for Hg^2+^ detection not only saves time but also improves the sensitivity of detection and simplifies the complexity of the experiment [64,65,66]. The reference electrode, working electrode, and counter electrode construct a three-electrode system in the electrochemical sensor [67] (Figure 1). The working electrode can be modified with different materials to specifically identify metal ion concentrations [68]. The presence of Hg^2+^ induces changes in current, potential, electrochemical impedance, capacitance, or electrochemiluminescence, enabling quantitative Hg^2+^ detection [69].

Many researchers often use DNA combined with graphene oxide (GO) to design a new electrochemical sensor for detecting metal ions (Table 2). This improves the sensitivity, selectivity, and even multi-path detection capabilities of the device [70]. GO-modified electrodes possess numerous advantages, including a large surface area, small volume, excellent electron transfer ability, and ease of surface modification [71,72]. Li et al. [73] used graphene in Nafion-G solution combined with in situ rhodium-plated electrodes to create an electrochemical sensor platform that enhanced signal detection. The study revealed that the composite membrane of Nafion-G showed highly sensitive detection for metal ions. This can interfere with the synergistic effect between graphene and Nafion. Noga Ratner et al. [74] developed a method for Hg^2+^ detection using indium tin oxide electrodes modified with gold nanoparticles based on glassy carbon, and the limit of detection (LOD) was 1 ng/L (5 × 10^−12^ M). Zhang et al. [75] combined graphene with DNA to design a faster and more sensitive electrochemical sensor to detect Hg^2+^. After the addition of Hg^2+^, the DNA sequence containing four thymine-thymidine (T-T) mismatches hybridized to the probe on the surface of the electrode with a T-Hg^2+^-T structure, increasing the peak current of [Ru(NH_3_)_6_]^3+^. The LOD in this method was 5.0 nM.

To improve the sensitivity of detection, researchers gradually used GO composite nanomaterials with DNA to detect Hg^2+^. Fang et al. [76] synthesized the microspheres in one step on reduced GO (Cu_2_OMS-rGO) and then placed them in an electrochemical sensor to detect Hg^2+^ in water, which would be rich in thymine (T). The single-stranded oligonucleotide was immobilized on the electrode modified with Cu_2_OMS-rGO. Hg^2+^ can cause thymine to mismatch with itself. This makes a “T-Hg^2+^-T” structure form. Then, the analyte Hg^2+^ and the complementary single strand were introduced. DNA, which caused the double-stranded DNA carrying Hg^2+^ to be immobilized on the composite electrode. The detection sensitivity of the Cu_2_OMS-rGO complex to Hg^2+^ was high, and the LOD was 8.62 pM.

Overall, our observations indicate that the combination of DNA and GO or GO complex has become a leading approach for Hg^2+^ detection. The unique properties of GO contribute to enhanced sensitivity and selectivity, thereby improving the overall effectiveness of detection methods.

## 3. Field Effect Transistors

FET is a biosensing platform that has attracted high attention in disease detection, such as urinary infections, human immunodeficiency virus (HIV) infections, and hepatitis B [77,78,79,80,81]. The system possesses the following advantages: high sensitivity, fast response, and inexpensive manufacturing process. FET sensor provides a sensitive detection for the reaction taking place on the surface of the gate electrode without a redox label [82]. In 2014, Knopfmacher et al. [83] reported a DNA probe-modified organic field effect transistor (OFET) sensor for detecting Hg^2+^ using the spin coating of gold nanoparticles (AuNPs) onto polymer OFETs. The thiolated probe was attached to the surface. After combining with Hg^2+^, the DNA formed a change in the charge density of the hairpin structure and indirectly detected Hg^2+^. This method truly opened the gate of the OFET-binding DNA sensor for detecting Hg^2+^, and the LOD was obtained at 10 μM. It is believed that under continuous optimization, it can quickly achieve extremely high detection sensitivity and become a biosensor with environmental benefits.

Recently, graphene field-effect transistors (GFETs) have attracted high interest in the field of sensors [84,85,86,87,88,89,90,91,92,93]. Tu et al. [94] prepared a liquid-controlled graphene field effect transistor (GFET) array biosensor (a 6 × 6 GFET on a chip) that can quantitatively detect Hg^2+^ using the aptamers with a LOD of 40 pM and a fast response time of less than one second. Wang et al. [95] developed a single-strand DNA containing four phosphorothioate-modified RNAs (Hg-DPR) to improve the yield of the cleavage reaction. The FET using single-walled carbon nanotubes (FETs/SWNTs) was made based on Hg-DPR. After exposure to Hg^2+^, Hg-DPR could be effectively cleaved, which further changed the SWNTs conductivity. The Hg-DPR/SWNTs/FET successfully detected Hg^2+^ with a LOD of 10 pM.

Overall, FET sensors can achieve extremely high detection sensitivity for Hg^2+^ with DNA. Two-dimensional materials can cause changes in electrical signals and enable the detection. This can be further used to improve the excellent sensing performance.

## 4. Raman Spectroscopy

Raman spectroscopy is essentially a scattering spectrum discovered by CV Raman from India. The molecular vibration and molecular structure can be obtained from the spectrum. After the information, the researchers discovered that their high sensitivity, non-invasive, non-marking, and fingerprinting advantages are widely used in daily life [96,97,98]. Raman plays an indispensable role in detecting Hg^2+^ with highly sensitive, quick, and efficient DNA. Uchiyama et al. [99] and Benda et al. [33] performed detailed Raman characterization of the T-Hg(II)-T base, revealing the effect of Hg^2+^ binding to DNA.

Surface-enhanced Raman scattering (SERS) is used by some researchers as a detection method (Table 3). Compared with ordinary Raman, SERS is an optimized detection method based on surface plasmon resonance. There is electron resonance between the surface plasmon and the light field [100]. Raman signal can be significantly enhanced by 10^14^ [101]. SERS is a promising method for detecting Hg^2+^ [102,103,104,105,106]. Some researchers have focused on the discovery of the “T-Hg^2+^-T method” and the interpretation of the SERS phenomenon. This method depends on T-Hg^2+^-T and has high selectivity and sensitivity [107,108,109,110,111,112,113]. Han et al. [113] double-labeled the 5′ and 3′ ends of DNA with thiol and tetramethylrhodamine (TAMRA), respectively. DNA could be connected to the surface of gold microshells spontaneously. Hg^2+^ detection was carried out with a LOD of 50 nM.

**Figure 2:**
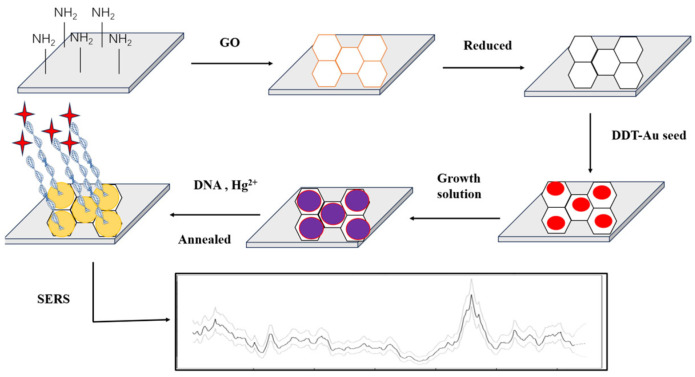
Preparation of gold nanoparticles (AuNPs)/reduced graphene oxide (rGO). SERS: surface-enhanced Raman scattering; DTT: DL-Dithiothreitol. The active substrates of heterojunction SERS and sensing for Hg^2+^. AuNPs were grown on the surface of rGO to form a SERS substrate, and the DNA probe was fixed with a thiol group using an Au-S bond. The detected trace of Hg^2+^ by T-Hg^2+^-T coordination reached the detection limit of 0.1 nM. Reproduced from Liu et al. (2013) with permission from the American Chemical Society [107].

Wu et al. [114] reported a SERS sensor for Hg^2+^ sensing. Most importantly, this SERS sensing strategy was not affected by external factors and showed good SERS sensing performance and repeatability. The aptamer sensor reached 0.4 pM for LOD. Wang et al. [115] modified Ag nanoparticles (AgNPs) with TAMRA functionalized aptamers. T-Hg^2+^-T resulted in the hairpin structure of the aptamer. AgNPs aggregated, and the SERS intensity increased significantly. To facilitate the fixation of the probe DNA on the surface of the noble metal, the probe was further modified with a thiol group. The LOD was 5 nM. Despite the high selectivity of these SERS sensors, most of the NPs in these sensors were prepared in the liquid phase; therefore, they were easily aggregated in an uncontrollable manner, which affected the repeatability of SERS detection [103]. Many noble metals, such as Au, Ag, Cu, etc., can produce strong electromagnetic enhancement effects, which are often used to develop substrates [116]. For example, Ding et al. [107] grew AuNPs on the surface of rGO to form a SERS substrate and fixed the DNA probe with a thiol group on the surface of Au. The traces of Hg^2+^ were realized with the LOD of 0.1 nM (Figure 2).

Liu et al. [117] developed a detection method using SERS. The method used a DNA molecular switch combined with a fragile silver film (Ag-film) to detect Hg^2+^ with high specificity. Single-stranded DNA was attached by the signal from a Raman probe with Hg^2+^. Due to the interaction between DNA bases and Hg^2+^, the specific structure of the DNA strand changed, and the signal was significantly enhanced. The SERS sensor achieved an ultra-low LOD (1.35 fM) of Hg^2+^ detection. Mohamed Shaban [118] invented a new type of SERS sensor, which functionalized the brass spiral nails through CoFe_2_O_4_ and grew carbon nanotubes (CNTs) on it. The Raman characteristic peak was enhanced 4 times, and the detection of Hg^2+^ with high sensitivity was realized through the adsorption of heavy metals with a range from 1 to 1000 ppb.

Cheng Tian et al. [119] reported a novel SERS sensing for trace Hg^2+^ using dual recycling amplification. During cyclic amplification, SERS is combined with a DNA amplification strategy to increase the Raman signal. When Hg^2+^ was added, the aptamer in the detection system formed a hairpin structure. Subsequently, through the role of polymerase, deoxy-ribonucleoside triphosphate (dNTPs) and Nicking endonuclease products from *Bacillus brevis* (Nt.BbvCI) in the reaction system, the trigger DNA could start the subsequent amplification reaction as a primer, and most SERS probes were stimulated to be fixed on the magnetic beads, which enhanced the Raman signal and improved the detection sensitivity with the LOD of 0.11 fM. Zhang et al. [120] developed a method for determining Hg^2+^. It is based on the principles of SERS and hybrid chain reaction (HCR). DNA is self-assembled on AuNPs to form a new signaling nanoprobe. The structure of T-Hg^2+^-T was fixed on magnetic beads. The HCR was initiated by the trigger DNA, which provided the binding sites to connect signaling nanoprobes. The sandwich structure was separated by using a magnetic field. The LOD was 0.08 pM.

Raman spectroscopy based on SPR provides a way to detect Hg^2+^ using the incident light frequency. Nanoparticles and other materials, such as carbon nanotubes, can improve the detection signals in Raman spectroscopy. DNA can also be amplified for higher signals for Hg^2+^. These two sides combine to further improve the detection sensitivity.

**Table 3:** Comparison of Raman spectroscopy-based Hg^2+^ detection biosensors.

DNA Probe	Nanomaterial or Other Auxiliary Material	Detection Linear Range	LOD	References
ssDNA	AuNPs/rGO/SiO_2_/Si heterojunction	0.1–6000 nM	0.1 nM	[107]
ssDNA	single gold micro-shell	0–10 μM	50 nM	[113]
dsDNA	Au@Ag NPs	0–200 nM	0.4 pM	[114]
ssDNA	AgNPs	0–25 nM	5 nM	[115]
ssDNA	Ag-film	0.1 pM–10 μM	1.35 fM	[117]
dsDNA	AuNPs	0.1 pM–10 nM	0.08 pM	[118]

AuNPs: Gold nanoparticles. rGO: Reduced graphene oxide. AgNPs: Silver nanoparticles.

## 5. Colorimetry

Colorimetric detection is a traditional detection method based on color reaction. The content and composition of the substance to be tested are determined by comparing and measuring the color depth of the solution in which the analyte is to be measured [121,122]. The advantages and disadvantages of colorimetric detection methods are obvious. On the one hand, it can allow real-time qualitative or semi-quantitative detection without complex instruments. On the one hand, due to the limited recognition of our naked eyes, the relative error of the results obtained is relatively large. Compared to methods for detecting heavy metal ions, the development of colorimetric detection methods is limited. However, with the development of biology and nanotechnology, new methods for designing colorimetric biosensors are emerging [123,124]. In the process, researchers have discovered that gold nanoparticles have a high extinction coefficient. The ideal color material for colorimetric sensor design, and more importantly, when the gold nanoparticles are close to each other and aggregate, the color of the nanoparticles can change, and nanomolar concentrations can be observed with the naked eye [125,126,127]. Thus, sensitive detection is performed with minimal material consumption [128].

Knecht et al. [129] used the surface plasmon resonance coupling effect of AuNPs combined with a DNA sensor to detect Hg^2+^. First, AuNPs were prepared using citrate [130], and then the principle of DNA hybridization and T-Hg^2+^-T was used to promote the aggregation of AuNPs with a detection limit of 100 nM. Wang et al. [131] proposed a biosensor for detecting Hg^2+^ using the bovine serum albumin-protected silver clusters (BSA-Ag NCs). BSA-Ag NCs were activated by Hg^2+^, and the surrounding dissolved oxygen was used as an oxidant. BSA-Ag NCs could be “activated” by Hg^2+^. The LOD was 25 nM. Tan et al. [132] reported a universal sensing for Hg^2+^ using the target-mediated AuNPs growth. First, 15T bases were used to detect Hg^2+^ using T-Hg^2+^-T coordination. The aptamer was desorbed from the surface of AuNPs after binding to Hg^2+^, and the rest of the aptamers underwent a morphological change, which caused the structure of AuNPs to change to form different colored solutions. In this case, the LOD was 9.6 nM. Both 25-mer and 59-mer aptamers had LODs of 4.05 nM and 3 nM.

Zhu et al. [133] proposed a new strategy to detect Hg^2+^ using a colorimetric method: aggregation of cationic polymer-driven AuNPs. In this three-component system, DNA was electrostatically bound to diethylene glycol diethyl diacrylate (PDDA) in an AuNPs solution. The hairpin structure induced by T-Hg^2+^-T was formed in the DNA strand and then no longer interacted with PDDA, so free PDDA promoted the aggregation of AuNPs. Therefore, a colorimetric sensor was established, and AuNPs were aggregated to detect Hg^2+^. The LOD for Hg^2+^ based on the naked eye was 5 nM, which was used for rapid monitoring of Hg^2+^. The LOD by UV-Vis spectroscopy was as low as 0.15 nM. The DNA/AuNPs-based colorimetric method is more expensive than using DNA. Febrina et al. [134] developed a lower-cost CA-AuNPs filter paper sensor to detect Hg^2+^ in water. Cyanuric acid (CA) is a compound that is similar to thymine in structure, which could form a CA-Hg^2+^-CA structure with Hg^2+^. Meanwhile, CA could stabilize AuNPs to prevent aggregation and then immobilize them on the surface of filter paper. When the CA-Hg^2+^-CA complexes were formed by Hg^2+^, the stability of CA-AuNPs was reduced, and AuNPs aggregation occurred subsequently. In addition to the simple T-Hg^2+^-T principle, there are other color change and detection principles successively applied to detect Hg^2+^ in colorimetric reactions. Shao et al. [135] reported a gold nanoparticle colorimetric probe (AuNPs) with treated N-methylpyridone and chloroauric acid (HAuCl_4_) as precursors that directly respond to Hg^2+^. When Hg^2+^ is present, AuNPs will gather, and the absorbance at 700 nm will increase. The color changes with the LOD of 0.3 μM.

Wang et al. [136] used single-stranded (ssDNA) paired valence thiol groups. Inhibition of the oxidase-like activity of the metal-organic framework (expressed as MVC-MOF), synthesis of MVC-MOF by partial oxidation of cerium (III) can produce Ce(IV) ions, which typically confers MVC-MOF is typically oxidase-like. ssDNA binds MVC-MOF to mask its active site, which inhibits catalytic activity. A colorimetric detection for Hg (II) was reported using thymidine-rich ssDNA (T-ssDNA) as the model DNA. Hg^2+^ binds to T-ssDNA to form T-dsDNA, which causes MVC-MOF to convert 3, 3′, 5, 5′-tetramethylbenzidine to a blue product with oxygen. The LOD was 10.5 nM.

Overall, colorimetric detection is a traditional detection method using color reaction. It is based on the change of light signals. These simple and effective colorimetric assays have gradually expanded the range of applications, such as molecular diagnostics, drug delivery, and environmental detection, which are increasingly important and have high application potential (Table 4).

## 6. Fluorescence Resonance Energy Transfer Detection

FRET detection is a method based on the principle of FRET, which is sensitive and efficient in detecting the object by the change of fluorescence intensity. It is the most widely used and most sophisticated method for detecting heavy metal ions [42,137,138,139]. The method not only has low cost, low consumables, high sensitivity, and high accuracy, but it also has a sufficient theoretical basis, and the prepared sensor has strong stability and high practical application efficiency [42] (Table 5). Hg^2+^ was detected by Ono and Togashi [33] using FRET. Zhan et al. [140] used FAM dye and ethynyl modification to detect Hg^2+^ at both ends of multiple T-base DNA strands. After the addition of Hg^2+^, the structure of the DNA sequence was changed, which resulted in a fluorescence energy transfer between FAM and ethynyl with a LOD of 16.15 nM. Buranacha et al. [141] used a new type of biosensor aptamer. This used two kinds of DNA, a FRET donor and an acceptor, to carry out fluorescence hybridization. This resulted in the hybridization of the two DNA strands after Hg^2+^ was added. The LOD was 7.037 ± 0.18 nM. Ren et al. [142] inserted two DNA intercalators, YOYO-1 and TOTO-1, into the DNA strand. After the addition of Hg^2+^, FRET occurred between the conjugated polymer (PFP) with a positive charge and the dye in the DNA strand with a negative charge. Therefore, the fluorescence intensity was increased by 37 times, and the LOD was 6 nM.

Most researchers designed the biosensor using the unique T-Hg^2+^-T structure to detect Hg^2+^ and obtained a good response. For example, the DNA sensor described above relates more or less to the specific structure of T-Hg^2+^-T. Zhou et al. [143] used 2-aminopurine (2AP) as a fluorescent marker in the middle of 10-mer DNA homopolymers. The 2-AP-labeled DNA provides an ultra-low background without the need for external quenching. The addition of Hg^2+^ reduces the accumulation of 2AP and its adjacent thymine, enhancing the fluorescence signal. Zhu et al. [144] released the Hg^2+^ according to the principle that the T-Hg^2+^-T structure was cleaved by the enzyme Exo III. The DNA sequence was reconstructed into a G4 structure to detect Hg^2+^, which contained specific binding of iridium (III). Sun et al. [145] designed a FAM-ssDNA probe using GO as a fluorescence quencher and exonuclease I to hydrolyze ssDNA. When GO is close enough to the fluorescent dye, the fluorescent dye can be quenched by FRET. Under the condition of adding Exo I, the ssDNA was hydrolyzed so that the fluorescent dye of FAM was released, and the fluorescence was restored. However, ssDNA could form double strands through the T-Hg^2+^-T construct after Hg^2+^ was added. This inhibited the activity of Exo I; fluorescence did not recover. The LOD was 3.93 nM.

Wu et al. [146] proposed a novel dual FRET system that can be used to detect both Pb^2+^ and Hg^2+^. The system used AuNPs as acceptors and two-color up-conversion nanoparticles (UCNPs) as donors. Therefore, the donor-acceptor pairs can be formed. Due to the good overlap between the spectrum of UCNPs and AuNPs, the green and red up-conversion fluorescence could be quenched. After adding Hg^2+^ and Pb^2+^, the aptamer preferentially selected the corresponding analyte binding and formed a hairpin-like structure of Hg^2+^. The FRET was disrupted, and the up-conversion fluorescence was restored. The fluorescence intensity increased with increasing metal ion concentration under optimized experimental conditions, and both Hg^2+^ and Pb^2+^ could be quantified. The LODs of the two were 150 pM and 50 pM. Li et al. [147] used a naphthalimide derivative (AHN) as a fluorescent label for thymidine (T)-rich ssDNA and Fe_3_O_4_ nanoparticle as a quencher. The labeled ssDNA was not separated from the magnetized GO without Hg^2+^, which resulted in the complete quenching of the fluorescence. The fluorescence recovery with the addition of Hg^2+^ was achieved away from GO fluorescence, with the LOD of 0.65 nM.

Hg^2+^ has a strong sulfophilicity, and a DNA sensor can be designed in a new direction according to this property. In 2015, Liu et al. [148] reported a mechanism of Hg^2+^-induced phosphorothioate (PS)-modified RNA cleavage in which a non-bridged oxygen atom in a DNA strand deoxynucleotide is replaced by a sulfur atom (S). Because Hg^2+^ is extremely sulfophilic, its binding to S leads to cleavage of the phosphate bond, causing the end with FAM to be far from the quencher, and the fluorescence intensity is enhanced. The study emphasizes that even if there is only a single bottom, the substance (without DNase) can also be cleaved by Hg^2+^. Zhao et al. [149] developed a ratio-metric fluorescent probe (namely APS-NA) with acridone as an energy donor and 1, 8-naphthalimide as an energy acceptor. When Hg^2+^ was present, the dithioacetal bond between acridone and 1, 8-naphthalimide, was destroyed. Then, the bright blue fluorescence was emitted, which achieved the detection of Hg^2+^. In addition, Liu et al. [150] developed a sensor using fluorescence quenching with PS-RNA chemistry and tested three DNA probes, each containing 1, 3, and 5 PS-RNA Hg^2+^ cleavage sites, demonstrating that increased cleavage sites will increase the Hg^2+^ cleavage rate relatively. Fluorescent-labeled Poly-A DNA was adsorbed on the surface of NGOs, and after the addition of Hg^2+^, the release of the fluorophore from GO resulted in enhanced fluorescence. The fluorophore-labeled DNA was used for detection, which has a slower response to Hg^2+^ and is susceptible to various external factors, while the PS-RNA probe is stable and has strong anti-interference ability. It is less affected by temperature and pH.

Overall, FRET can be carried out to detect the object. The effect of quenching is higher, and the sensitivity of detection is usually higher. Therefore, more quenchers with low cost have been used to improve the effect of quenching.

## 7. Conclusions

Given the wide range of techniques available for Hg^2+^ sensing, it is crucial to thoroughly understand their respective strengths and weaknesses to select the most appropriate detection strategy. Therefore, in addition to a detailed discussion of the key features of each technique, a comparative table is presented to provide a clearer evaluation of their advantages and limitations (Table 6).

If the sensitivity of outdoor detection is not high, the electrochemical sensors are more suitable for point-of-care diagnosis because of the relatively low detection cost and easy-to-obtain direct detection results. Raman spectroscopy and electrochemical sensors are limited in the number of samples they can detect or are unable to detect multiple samples simultaneously. Given these issues, there is a continuing need to explore more stable, sensitive, low-cost, efficient, and time-efficient methods for detecting Hg^2+^. Advancements in this area are crucial not only for environmental protection but also for ensuring human health.

## Figures and Tables

**Table 1:** Recently published reviews on similar topics.

Title	Topic	Year	References
Gold nanoparticle probes for the detection of mercury, lead, and copper ions	Gold nanoparticle probes	2011	[48]
Colorimetric detection of mercury ions based on plasmonic nanoparticles	Colorimetric detection	2013	[49]
Highly sensitive fluorescence detection of mercury (II) ions based on DNA machine amplification	Fluorescence detection	2013	[50]
SERS-based mercury ion detections: principles, strategies and recent advances	SERS-based mercury ion detections	2016	[51]
Simultaneous detection and determination of mercury (II) and lead (II) ions through the achievement of novel functional nucleic acid-based biosensors	Nucleic acid-based biosensors	2018	[52]
The detection of mercury ions using DNA as sensors based on fluorescence resonance energy transfer	Fluorescence resonance energy transfer	2019	[53]
Nanozyme-based sensing platforms for detection of toxic mercury ions: An alternative approach to conventional methods	Nanozyme-based sensing	2020	[54]
Recent progress in functional materials for selective detection and removal of mercury (II) ions	Functional materials	2020	[55]
Recent progress in nanoparticles-based sensors for the detection of mercury (II) ions in environmental and biological samples	Nanoparticles based sensors	2020	[56]
Small molecular fluorescent probes for the detection of lead, cadmium and mercury ions	Small molecular fluorescent probes	2021	[57]
Construction of DNA biosensors for mercury (II) ion detection based on enzyme-driven signal amplification strategy	Enzyme-driven signal amplification strategy	2021	[58]
Fluorescent and colorimetric sensors for the detection of lead, cadmium, and mercury ions	Fluorescent and colorimetric sensors	2022	[59]
Review on fluorescent sensors-based environmentally related toxic mercury ion detection	Fluorescent sensors	2022	[60]
Recent advances in fluorescent materials for mercury(ii) ion detection	Fluorescent materials	2023	[61]
DNA sensors for the detection of mercury ions	DNA sensors	2025	This work

**Table 2:** Comparison of electrochemistry-based Hg^2+^ detection biosensors.

DNA Probe	Nanomaterial or Other Auxiliary Material	Detection Linear Range	LOD	References
ssDNA	Au	10 pM (1.1 ppt)–500 nM (56.2 ppb)	10 pM	[62]
ssDNA	AuNPs	1 × 10^−12^–1 × 10^−8^ M	0.69 pM	[64]
ssDNA	bisferrocene	1–625 pM	0.6 pM	[65]
dsDNA	Pd@Cu@Pt MMN/bisferrocene	10 fM–100 nM	3.58 fM	[66]
ssDNA	GO	8.0 × 10^−9^–1.0 × 10^−7^ M	5.0 × 10^−9^ M	[75]
ssDNA	Cu_2_OMS–rGO	0.05–40 nM	8.62 pM	[76]

**Table 4:** Comparison of colorimetry-based Hg^2+^ detection biosensors.

DNA Probe	Nanomaterial or Other Auxiliary Material	Detection Linear Range	LOD	References
dsDNA	AuNPs	0–2 μM	100 nM	[129]
ssDNA	BSA-Ag NCs	80 nM–50 mM	25 nM	[131]
ssDNA	AuNPs	0–60 nM	9.6 nM	[132]
		10–150 nM	4.05 nM	
		5–40 nM	3.0 nM	
ssDNA	AuNPs	0.25–500 nM	0.15 nM	[133]
T-dsDNA	MVC-MOF	0.05–6 μM	10.5 nM	[134]

AuNPs: Gold nanoparticles. BSA-Ag NCs: BSA-capped silver nanoclusters. MVC-MOF: Mixed-valence state cerium-based metal-organic framework.

**Table 5:** Fluorescence resonance energy transfer sensors for Hg^2+^ detection.

DNA Probe	Nanomaterial or Other Auxiliary Material	Detection Linear Range	LOD	References
ssDNA	FAM dye + ethynyl modification	16.1–1495.5 nM	16.15 nM	[140]
Aptamer	FRET donor/acceptor DNA pairs	7.03 ± 0.18 nM	7.037 ± 0.18 nM	[141]
dsDNA	YOYO-1, TOTO-1 + PFP polymer	6–10 nM	6 nM	[142]
2AP-labeled DNA	10-mer DNA homopolymers	8.4–600 nM	8.4 nM	[143]
G4 DNA	Exo III enzyme + Ir(III)	1.5–5 nM	1.5 nM	[144]
ssDNA	GO + Exonuclease I	0–250 nM	3.93 nM	[145]
Aptamer	AuNPs + UCNPs (dual FRET)	0.5–500 nM	150 pM	[146]
ssDNA	AHN + Fe_3_O_4_ NPs + magnetized GO	1–10 nM	0.65 nM	[147]
PS-RNA	Sulfur-modified RNA + FAM	0–1 μM	1.7 nM	[148]
APS-NA	Acridone + 1,8-naphthalimide	0–30 μM	1 μM	[149]
PS-RNA	NGOs + GO	8.5 nM	8.5 nM	[150]

**Table 6:** Advantages and limitations of Hg^2+^ detection techniques.

Techniques of Hg^2+^ Sensing	Advantages	Limitations	References
Electrochemistry	High sensitivity, rapid response, fast analysis, cost-effective, portable, and compatibility with portable devices	Complexity stability requires electrode modification, possible interference from other metal ions, and complex sample preparation.	[151,152]
Field Effect Transistors (FET)	Capable of detecting trace amounts of Hg^2+^ due to changes in the electrical properties of the transistor, high-sensitivity, and portable biochemical detection platform	Susceptible to contamination and non-specific adsorption, which may reduce sensitivity, structures are vulnerable to damage during multistep post-production treatment and weak interactions between the electrodes and semiconductor layers.	[153,154]
Raman spectroscopy	High molecular specificity, non-destructive analysis, potential for multiplex detection.	Low sensitivity, need for signal enhancement (e.g., SERS), and expensive instrumentation.	
Colorimetry	Simple, cost-effective visual detection without specialized equipment.	Limited sensitivity and selectivity, potential interference from colored substances.	[155]
Fluorescence Resonance Energy Transfer (FRET)	Enhances signal rationing and the sensitivity of the method, as well as real-time detection and capability for in vivo monitoring.	Fluorophore instability, photobleaching, and the need for careful probe design.	[53,156]
